# World Kidney Day as a Platform for Action: Early Outcomes of a Vascular Access Mission in Sub-Saharan Africa

**DOI:** 10.7759/cureus.106209

**Published:** 2026-03-31

**Authors:** Oumarou Moussa, Gaya Hamza, Etok Ariane, Chime Sandrine, Tsambang Lionel, Iya Atikou, Djidjoua Durand, Sango Sango, Kobe Fokalbo, Neossi Mathurin

**Affiliations:** 1 Department of Internal Medicine, Faculty of Medicine and Biomedical Sciences, University of Garoua, Garoua, CMR; 2 Department of Nephrology, Ngaoundéré Regional Hospital, Ngaoundéré, CMR; 3 Department of Surgery, Ngaoundéré Regional Hospital, Ngaoundéré, CMR; 4 Department of Surgery, Faculty of Medicine and Biomedical Sciences, University of Garoua, Garoua, CMR; 5 Department of Internal Medicine, Faculty of Medicine and Pharmaceutical Sciences, University of Douala, Douala, CMR; 6 Department of Internal Medicine, Ngaoundéré Regional Hospital, Ngaoundéré, CMR; 7 Department of Surgery, Douala General Hospital, Douala, CMR; 8 Department of Surgery, Yaounde General Hospital, Yaounde, CMR; 9 Department of Radiology, Ngaoundéré Regional Hospital, Ngaoundéré, CMR

**Keywords:** arteriovenous fistula, cameroon, global health equity, hemodialysis, implementation science research, resource-limited settings, vascular access, world kidney day

## Abstract

Background and aim

Resource-limited settings in sub-Saharan Africa face critical barriers to arteriovenous fistula (AVF) creation, forcing hemodialysis patients into prolonged dependence on catheters, with the associated risks of infection and increased mortality. Ngaoundéré, a referral city in northern Cameroon, launched chronic hemodialysis services in February 2023 but lacks a resident vascular surgeon. Consequently, nearly all incident patients initiate hemodialysis using temporary central venous catheters (CVCs), a practice associated with well-documented complications. This retrospective analysis evaluates the outcomes of a Vascular Access Surgical Mission implemented over three annual cycles (March 2023 to March 2025) synchronized with World Kidney Day events. The primary objectives were to assess the feasibility of AVF creation and the rate of functional maturation at three months. Secondary objectives included evaluating procedural safety, early complication rates (thrombosis and stenosis), the reduction in catheter-associated bloodstream infections, achievement of catheter independence, and patient-reported quality of life. An exploratory, hypothesis-generating analysis was also conducted to identify potential risk factors associated with maturation failure.

Methods

We retrospectively reviewed clinical records of 35 adult patients who underwent AVF creation at Ngaoundéré Regional Hospital during three surgical missions (March 2023, March 2024, and March 2025). Data were extracted from pre-operative assessments, hemodialysis files, post-operative records, and ultrasound reports. Primary outcomes were AVF creation feasibility and functional maturation (per Kidney Disease Outcomes Quality Initiative {KDOQI} criteria) at three months. Secondary outcomes included procedural safety, early complications, and infection rates. Historical comparator data were reviewed for infection comparisons. Patient satisfaction was assessed via a custom Likert-scale questionnaire.

Results

Of 35 enrolled patients (mean age 40±12 years; 63% male), 34 AVFs were successfully created (97.1%, 95% CI: 85.1-99.5%). Functional maturation occurred in 29 of 32 patients who completed the three-month follow-up (90.6%, 95% CI: 75.8-96.8%). One early thrombosis (2.9%) was successfully salvaged; two cases of early stenosis (5.9%) required revision. No catheter-associated bloodstream infections (CABSI) occurred in AVF users (0 per 1,000 AVF-days) compared with 5.0 per 1,000 catheter-days in the historical comparator. Catheter dependence fell from 100% to 12.5% at three months. Patient satisfaction was high (92% reported reduced anxiety regarding infection; 94% would recommend the program). These early maturation rates provide a foundation for assessing long-term durability; 12- and 24-month patency data are currently being collected and will be critical for establishing sustained clinical benefit.

Conclusions

When combined with point-of-care imaging, equity-focused patient prioritization, and structured follow-up, mission-based surgical delivery can achieve high rates of AVF creation and functional maturation, with early outcomes comparable to those reported in high-resource settings in the context of a feasibility study. However, the model as implemented represents a hybrid approach requiring continued external surgical expertise rather than fully independent local capacity. Long-term patency validation and sustained investments in local capacity are essential.

## Introduction

Chronic kidney disease is a major public health concern worldwide [1]. It progresses insidiously to end-stage kidney disease (ESKD), at which point renal replacement therapies become necessary. These modalities include conservative medical management, kidney transplantation, peritoneal dialysis, and hemodialysis (HD) [2]. Although hemodialysis remains the most common modality globally, patients with end-stage renal disease who require HD face a challenging reality as follows: it imposes various conditions on the patient, especially the need for vital vascular access to enable connection to the dialysis machine [2]. In 2020, the National Kidney Foundation (NKF) released the Kidney Disease Outcomes Quality Initiative (KDOQI) Clinical Practice Guideline for Vascular Access, which strongly recommends a functional arteriovenous fistula as the cornerstone for hemodialysis vascular access because of its lower infection rates, superior patency, and improved patient survival compared with other strategies [3]. In Africa, the burden of chronic kidney disease (CKD) and end-stage renal disease (ESRD) is rapidly increasing, fueled by hypertension and diabetes and exacerbated by limited primary care access and late referral [4]. Patients are most often seen at an advanced stage of their kidney disease, and late referral remains the norm [5]. This implies that incident hemodialysis patients are placed on a temporary dialysis catheter and face significant mortality in dialysis centers. Furthermore, catheter-related infection is the leading cause of death among dialysis patients in some areas of developing countries [6]. For patients seen early enough, the scarcity of dedicated vascular surgeons, as well as geographic and socioeconomic barriers, continually reduces the chances of obtaining the recommended vascular access. In Cameroon, nephrology and hemodialysis services have been established, but show marked disparities between urban centers and rural areas [7]. The northern Sahelian region of the country epitomizes this geographic and structural inequity. Ngaoundéré, the capital city of the Adamawa Region and the only nephrology referral center in northern Cameroon with a permanent nephrologist, began chronic hemodialysis services in February 2023, but the center lacks a dedicated vascular surgeon, an interventional radiologist, or a formal vascular access program. Consequently, temporary non-tunneled central venous catheters (CVCs) have become the default access modality for hemodialysis, a practice associated with up to 35-fold higher bacteremia rates and substantially reduced survival compared with arteriovenous fistula (AVF) users [8]. Financial and geographic barriers further exacerbate this inequity as follows: patients must travel >1,000 km (approximately 18-24 h by road or 14-16 h by train) to Yaoundé (the political capital of Cameroon) or Douala (the economic capital of Cameroon) with typical bus or train fares ranging from 10,000-20,000 CFA (~$16-32) one-way; though total costs including accommodation, meals, and surgical fees often exceed $400-450, representing five to eight months of median household income in the region: an insurmountable barrier for most economically disadvantaged families [9,10]. Even when AVFs are successfully created, patients’ follow-up or reinterventions are often limited or impossible, compromising long-term functionality [11]. To address this gap without requiring patients to cross borders, we designed and implemented a vascular access surgical mission, strategically aligned with World Kidney Day (WKD), to provide functional AVFs to our patients. This retrospective cohort study describes the design, execution, outcomes, and implications across three missions (March 2023 to March 2025), and provides insights into vascular access delivery in rural sub-Saharan Africa. The primary objectives were to assess the feasibility of AVF creation and the rate of functional maturation at three months. Secondary objectives included evaluating procedural safety, early complication rates (thrombosis and stenosis), the reduction in catheter-associated bloodstream infections, achievement of catheter independence, and patient-reported quality of life. An exploratory, hypothesis-generating analysis was also conducted to identify potential risk factors associated with maturation failure.

An abstract of this work has been submitted to the forthcoming World Congress of Nephrology in Yokohama, Japan, scheduled for late March 2026. The abstract has been accepted and will be presented at the Congress.

## Materials and methods

Study design and rationale

The vascular surgery mission was designed as a model in which a specialist vascular surgeon travels from major cities of the country to the city of Ngaoundéré. This model addresses the following three interconnected issues: no vascular surgeon is available within 1,000 km, transportation and surgery cost more than five to eight months of a dialysis patient’s family income, and the hospital lacks an official protocol for vascular access.

Design principles

The missions take place annually in mid-March, coinciding with World Kidney Day celebrations to garner support from local and national institutions and to leverage this platform to highlight the importance of vascular access in hemodialysis [12]. Support has been obtained from the Cameroonian Society of Nephrology and from visiting volunteer vascular surgeons from Yaoundé and Douala. Another principle was the adaptation of local resources and the strengthening of the hospital’s skills as follows: the operations took place in the hospital’s minor operating theater with local surgeons as assistants [13]. Medical students in their final year and dialysis nurses participated as observers and assistants.

Mission duration and structure

Each mission lasted five days and was organized from Wednesday to Sunday. The first day was dedicated to refresher training for local staff with PowerPoint (Redmond, WA: Microsoft Corp.) presentations in the dialysis conference center on vascular anatomy, arteriovenous fistulas, and, for nurses, particular emphasis on post-operative monitoring and the management of complications. The afternoon was devoted to meetings with selected patients for clinical examination, vascular ultrasound, and patient education. Days two to three were dedicated to the surgical operations (10-12 cases per mission). Days four to five were reserved for post-operative monitoring, training and feedback workshops, and information sharing.

Study context

The Ngaoundéré Regional Hospital (population around 220,000) was established in 1934 and is the largest public hospital in the Adamawa Region (population 1.2 million). It houses the region’s only hemodialysis unit. The center was inaugurated by the Minister of Public Health in December 2022 and began operations on January 31, 2023. At its inauguration, the center had 10 dialysis machines and has seen a steady increase in its active patient caseload, rising from an average of 15 patients per month in 2023 to an average of 40 patients per month in 2025. Since opening, the team has included one nephrologist, eight dialysis nurses, one biomedical technician, and no vascular surgeon or interventional radiologist on site.

Data collection

The data and variables for analysis were collected from pre-operative clinical evaluation forms, operative notes, dialysis medical records, Doppler ultrasound reports, and administrative records of the dialysis unit. All available data were entered into a standard electronic form with regular checks to ensure completeness. An independent reviewer (a final-year medical student) randomly checked 10% of the forms to verify the accuracy of data entry. Agreement among data entry personnel exceeded 95% for critical information. This study was approved by the Ethics Committee of Ngaoundéré Hospital and complied with the Declaration of Helsinki and the Strengthening the Reporting of Observational Studies in Epidemiology (STROBE) guidelines for cohort studies.

Patient selection and prioritization eligibility criteria

Inclusion criteria included age ≥18 years, stage 5 chronic kidney disease (CKD) on chronic hemodialysis for ≥4 weeks via temporary CVC, no prior AVF attempt, adequate vessels on ultrasound (artery ≥2.0 mm, vein ≥2.5 mm), and no contraindication to local anesthesia. Exclusion criteria were active infection, severe heart failure (New York Heart Association {NYHA} class IV), and life expectancy <3 months, acute kidney injury, or inability to provide informed consent. Because demand exceeded capacity, an equity-based points system was used for prioritization (maximum eight points) as follows: age <50 years (two points), socioeconomic vulnerability defined as unemployment or a monthly income <$40 (two points), absence of major comorbidities (two points), and residence in the Adamawa Region or <400 km from Ngaoundéré (two points). Patients were ranked according to a score (maximum eight points). Those with the highest score (6-8 points) were given priority. The scores were verified by final-year medical students; in case of disagreement, the local nephrologist had the final say. Socioeconomic vulnerability was verified through a direct household income assessment conducted by the hospital social worker during the screening interview. Patients were classified as "vulnerable" if their monthly household income was below the national poverty line ($40/month) or if they reported being unemployed and having no other household member in formal employment. Geographic accessibility was assessed based on patients' district of residence; priority was given to patients residing within the Adamawa region to ensure feasible follow-up. The prioritization framework was applied prospectively by a multidisciplinary committee comprising the nephrologist, a social worker, and the visiting surgeon. Final selection was determined by consensus, with explicit documentation of the rationale for inclusion.

Pre-operative assessment

All patients underwent a standard assessment, including clinical evaluation, dialysis quality (Kt/V >1.2), and on-site Doppler ultrasound (point-of-care ultrasound {POCUS}) [14]. The selection criteria were artery diameter ≥2.0 mm and vein diameter ≥2.5 mm (before compression), compressibility, and freedom from clots. The examination protocol followed standard criteria: arterial diameter was measured in the transverse plane at the site of planned anastomosis; venous diameter was measured with a tourniquet applied to the upper arm to simulate distension, following a 2-min period of dependency. Contextual adaptations to KDOQI maturation criteria as follows: (1) substitution of transit-time flow measurement with Doppler-derived flow volume due to equipment constraints, (2) acceptance of palpable thrill + audible bruit as sufficient clinical confirmation of maturation in the absence of routine angiography, (3) definition of "successful cannulation" as two consecutive sessions with 17-18G needles without infiltration, acknowledging variable nurse experience levels. These adaptations were consensus-derived by our local team and are now explicitly reported to support reproducibility. Standard laboratory tests were performed. Patients on anticoagulants stopped their medication two days before the operation. All results were reviewed jointly by the surgeon and nephrologist to choose the best site (radiocephalic preferred if possible). The vascular ultrasound assessment tool was prospectively designed in January 2023, prior to the first surgical mission.

Surgical equipment and technique

Standard equipment adapted to local resources was used. Anesthesia was administered with 2% lidocaine without epinephrine [15]. The surgical technique was an end-to-side anastomosis (radiocephalic or brachiocephalic) with a duration of approximately 1 h. All equipment was available from the hospital pharmacy or local suppliers. Patients were discharged the same day after confirming the presence of a thrill.

Post-operative follow-up and maturation criteria

The follow-up included clinical examination and day one vascular Doppler ultrasound, routine consultation on day seven, and complete assessment at weeks four and 12. Maturation criteria (KDOQI 2019, adapted to the local context) included palpable thrill, audible bruit, vein ≥6 mm, flow rate ≥500 mL/min, and successful cannulation with 17-18 gauge needles during two consecutive sessions. Complications were defined as early failure (non-maturation or thrombosis <24 h), primary patency (defined as the interval from AVF creation to any intervention for thrombosis or stenosis), primary assisted patency (including prophylactic revisions), and secondary patency (including salvage procedures after thrombosis), with timepoints clearly defined at one, three, and 12 months (not reported here). The three-month "functional patency" reported in this analysis corresponds to secondary patency with successful cannulation, per KDOQI operational definitions.

Statistical methods

Data were entered into a secure database (Microsoft Excel 2019; Redmond, WA: Microsoft Corp.). SPSS software (Armonk, NY: IBM Corp.) was used for the analyses. Given the small sample size, confidence intervals are wide. Continuous variables were assessed for normality using the Shapiro-Wilk test. Normally distributed variables are presented as mean±standard deviation. Non-normally distributed variables are presented as median (interquartile range). Significance level was set at α=0.05 (two‑tailed). Exact confidence intervals were calculated for binary results. Univariate analysis of factors associated with maturation failure was performed. Results are presented with odds ratios and confidence intervals [16].

Patient satisfaction assessment

Patient satisfaction was assessed using a structured Likert-scale questionnaire administered by trained dialysis nursing staff at the three-month follow-up visit. The instrument consisted of four items as follows: (1) Has your anxiety regarding infection changed since AVF creation? (five-point scale - much improved, improved, no change, worsened, much worsened); (2) How would you rate your overall quality of life since AVF creation? (five-point scale - much improved to much worsened); (3) Would you recommend this vascular access program to other dialysis patients? (yes/no); and (4) Are you satisfied with your current access to hemodialysis? (five-point scale). Individual responses were recorded; responses of "improved" or "much improved" were counted as positive. This questionnaire was not formally validated in other populations but was pilot-tested with five dialysis patients prior to implementation for comprehension and acceptability. The complete questionnaire (English and French versions) is provided in the appendix.

Historical comparator infection data collection

The historical comparator infection data were obtained from a retrospective institutional audit of the Ngaoundéré Regional Hospital dialysis unit for all patients maintained on non-tunneled CVC-only dialysis during January-December 2024. Data extraction methods included as follows: (1) review of laboratory microbiology records for all positive blood cultures; (2) cross-reference with clinical infection documentation in dialysis medical records; (3) classification by infection type, date of onset, and organism identified; and (4) calculation of catheter-associated bloodstream infections (CABSI) per 1,000 catheter-days. Infection surveillance definitions and methods were standardized across both cohorts. CABSI was defined according to CDC criteria as bacteremia in a patient with a central venous catheter, without a source of infection elsewhere. Data were extracted by a single reviewer (a final-year medical student) using a standardized form and were verified by the nephrologist for accuracy. While infection surveillance definitions were consistent between cohorts, temporal differences in care standards, documentation practices, and antibiotic regimens between the 2023-2025 mission period and the January-December 2024 historical audit period represent a limitation of this cross-cohort comparison, which we acknowledge.

## Results

Patient flow and baseline characteristics

The patients were relatively young (mean age of 40 years) and predominantly male (Table 1). Hypertension was the leading cause of chronic kidney disease (68.6%), consistent with epidemiological patterns in sub-Saharan Africa, where hypertension represents the primary etiology of end-stage renal disease (ESRD) [17]. The etiology of end-stage renal disease was determined by clinical assessment and documented from dialysis intake records. Kidney biopsy confirmation was not performed, consistent with standard practice in our resource-limited setting. Pre-operative hemoglobin was low (8.9±1.4 g/dL), typical for patients on maintenance hemodialysis. In this cohort, patients had been on CVCs for a median time of six weeks (IQR: 4-8), with 31.4% having prior CVC-related infection. The socioeconomic study revealed that 51.4% were unemployed, 22.9% had informal jobs, and the mean household income was only $38/month, below regional averages. Vessel mapping showed 74.3% with suitable radiocephalic vessels, 22.9% requiring a brachiocephalic approach, and 2.9% with no suitable vessels.

**Table 1:** Baseline demographic, clinical, socioeconomic, and vessel mapping characteristics of 35 patients who underwent AVF creation during the surgical missions (March 2023-March 2025). *BMI calculated as dry weight measured at routine dialysis session. AVF: arteriovenous fistula; CVC: central venous catheter

Variables		Value (n=35)
Demographics	Age (years), mean±SD	40.3±11.8
	Male sex, n (%)	22 (62.9)
	Body mass index (kg/m²), mean±SD	21.4±3.6*
Etiology of end-stage renal disease	Hypertension, n (%)	24 (68.6%)
	Diabetes mellitus, n (%)	6 (17.1%)
	Glomerulonephritis, n (%)	3 (8.6%)
	Unknown etiology, n (%)	2 (5.7%)
Dialysis characteristics	Dialysis vintage (days), median (interquartile range)	92 (65-120)
	Hemoglobin at baseline (g/dL), mean±SD	8.9±1.4
	Central venous catheter duration (weeks), median (interquartile range)	6 (4-8)
	Prior CVC-related bacteremia, n (%)	11 (31.4%)
Socioeconomic characteristics		
Employment status	Formal employment, n (%)	9 (25.7%)
	Informal employment, n (%)	8 (22.9%)
	Unemployed, n (%)	18 (51.4%)
	Monthly household income, USD	38±11
Pre-operative vessel mapping	Radiocephalic AVF suitable, n (%)	26 (74.3%)
	Brachiocephalic AVF suitable, n (%)	8 (22.9%)
	No suitable vessels, n (%)	1 (2.9%)

Patient flow and outcomes

**Figure 1:**
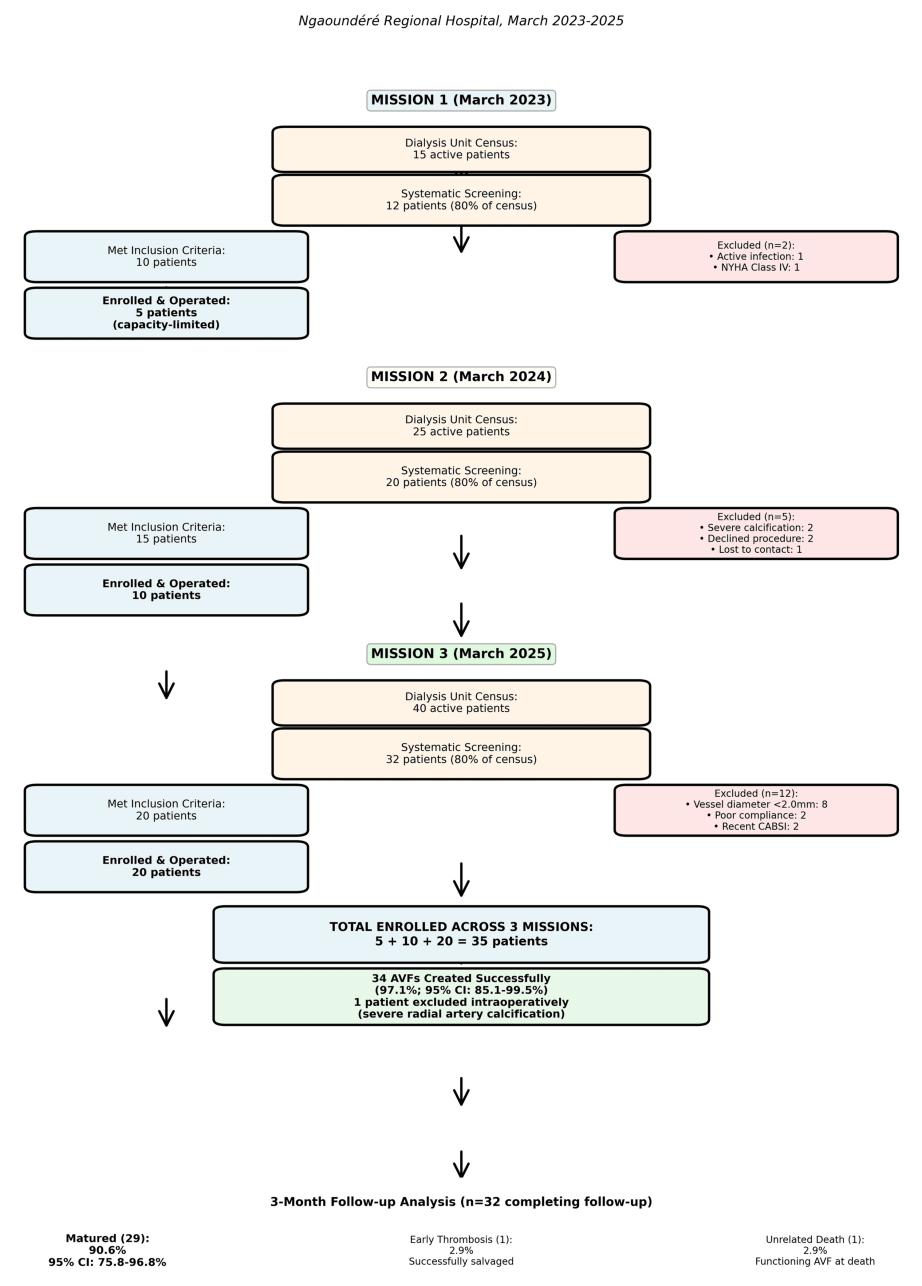
Patient flow diagram of the vascular access surgical mission. AVF: arteriovenous fistula; NYHA: New York Heart Association

Of the active dialysis census (n=84 across missions), 67 patients (80%) were screened, with 50 patients meeting the inclusion criteria and 35 patients enrolled and undergoing surgery (34 AVFs created). The remaining 20% were not screened due to logistic constraints (mission timing conflicting with patient schedules) or clinical exclusions (acute illness precluding assessment), potentially introducing selection bias toward more stable and engaged patients. The patient flow diagram of the mission is given in Figure 1.

**Figure 2:**
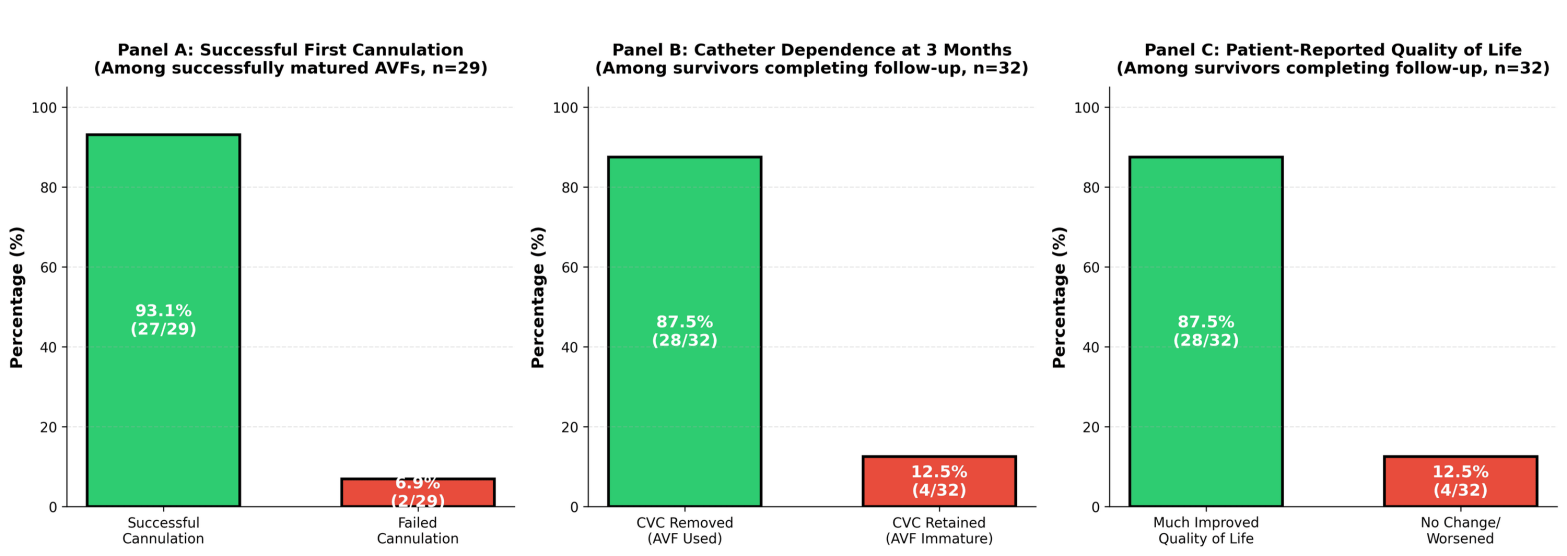
Secondary outcomes: AVF maturation, catheter independence, and patient-reported quality of life (all bar labels included both percentages and denominators {n} for standalone interpretability). AVF: arteriovenous fistula; CVC: central venous catheter The total number of patients is denoted by "n" with available data for each outcome; denominators vary due to follow‑up completion and survival.

One patient was excluded intra-operatively because of severe radial artery calcification discovered during dissection, preventing safe anastomosis. Thirty-two patients completed the three-month follow-up (one patient died at six weeks post-procedure from progressive heart failure {NYHA class IV}, adjudicated by the local nephrology team as unrelated to the AVF procedure or vascular access status, based on clinical trajectory, absence of procedural complications, and independent review). Of the 35 enrolled patients, 34 AVFs were successfully created (97.1%, 95% CI: 85.1-99.5%) with 25 radiocephalic (73.5%) and nine brachiocephalic (26.5%). The mean operative time was 42.1±8.8 min (range: 28-67 min; radiocephalic 38±7 min, brachiocephalic 51±6 min). No intra-operative complications (blood loss requiring transfusion, nerve injury, arrhythmia, or anesthetic adverse events) occurred. All 34 patients were able to ambulate within 2 h and were discharged the same day. At three months, 29 of the 32 remaining patients achieved functional patency (90.6%, 95% CI: 75.8-96.8%) with successful first cannulation in 27 patients (93.1%), a mean flow volume of 620±110 mL/min was measured via Doppler ultrasound at the four-week post-operative assessment (range: 480-850 mL/min), and a median time to first cannulation of 34 days (IQR: 28-41). Before every mission, the cohort used central vascular catheters (median duration 6.2 weeks). At three months post-AVF, 28/32 surviving patients (87.5%) successfully discontinued CVC use. No CABSI occurred in the AVF group (0 per 1,000 AVF-days), and two CABSI episodes were documented among the four catheter-dependent patients during the same study period (50% incidence). We also reviewed infection records from the Ngaoundéré dialysis unit for 187 patients maintained on CVC-only dialysis during January-December 2024 (unpublished institutional audit) and found that CABSI incidence in the historical CVC cohort was 5.0 episodes per 1,000 catheter-days. Infection surveillance methods and CABSI definitions (per CDC criteria - fever, positive blood culture without other source) were consistent between the historical audit (chart review of CVC-only patients) and the mission cohort. Primary outcomes are summarized in Table 2 and illustrated in Figure 2.

**Table 2:** Primary and secondary outcomes. *Denominator excludes one patient excluded intra-operatively, one death unrelated to AVF, and one lost to follow-up. Results include two patients awaiting maturation completion and two with late thrombosis managed with interventional thrombectomy. †Among successfully cannulated AVF (n=29). CABSI: catheter-associated bloodstream infection; QoL: quality of life; VASM: vessel assessment and selection mapping; AVF: arteriovenous fistula

Outcomes	Results
Primary outcomes	
AVF creation feasibility	34/35 (97.1%; 95% CI: 85.1-99.5%)
AVF maturation within 4 weeks*	31/33 (93.9%; 95% CI: 80.4-98.3%)
Functional patency at 3 months*	29/32 (90.6%; 95% CI: 75.8-96.8%)
Secondary outcomes	
Successful cannulation on first attempt	27/29 (93.1%)
Mean flow volume, mL/min†	620±110 (range: 480-850)
Median time to first cannulation (days), median (interquartile range)	34 (28-41)
Catheter-related outcomes	
Catheter dependence at 3 months	4/32 (12.5%)
Successful catheter removal	28/32 (87.5%)
New CABSI in AVF group (0-3 months)	0/28 (0.0%)
New CABSI in catheter group (0-3 months)	2/4 (50.0%)
Patient-reported outcomes	
Patients reporting "much improved" QoL	28/32 (87.5%)
Would recommend VASM to others	30/32 (93.8%)

Univariate risk factor analysis (hypothesis-generating)

This section presents univariate, hypothesis-generated associations only because, with only four maturation failures among 32 follow-up-eligible patients at three months, multivariate regression analysis cannot be performed. These findings should be considered as exploratory observations only (Table 3).

**Table 3:** Exploratory univariate analysis: hypothesis‑generating only due to small number of events (n=4). AVF: arteriovenous fistula

Risk factor	OR	95% CI	p‑Value
Female sex	7.88	0.72-86.2	0.089
Diabetes mellitus	6.25	0.80-48.9	0.077
Vein diameter <2.5 mm	13.5	1.45-125.6	0.021

**Figure 3:**
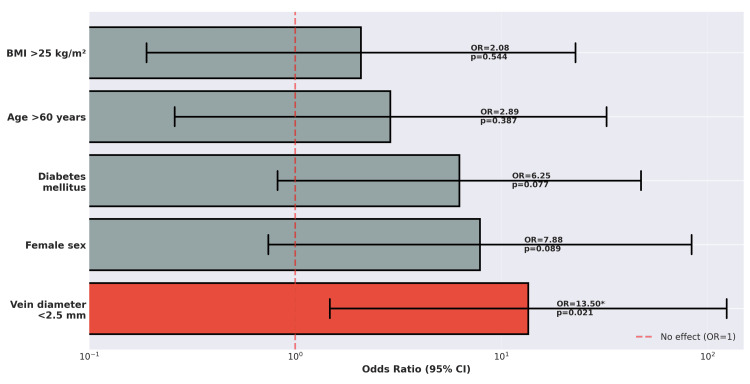
Risk factors for AVF maturation failure. Univariate analysis (n=33 eligible). *P<0.005 was statistically significant. Note: wide confidence intervals reflect small sample size (n=4 maturation failures). Findings are hypothesis-generating. AVF: arteriovenous fistula

Univariate analysis of risk factors for maturation failure is presented in Figure 3. Preliminary exploration of potential risk factors for maturation failure (n=4 failures among 34 AVFs) identified in univariate analysis (p<0.10) as follows: female sex (OR: 7.88), diabetes (OR: 6.25), and vein diameter <2.5 mm (OR: 13.5) as main factors. The extremely wide confidence intervals (all crossing 1.0) and small number of failures (n=4) indicate high instability. These hypotheses may reflect findings from a small sample with limited statistical power or confounding by other unmeasured variables. At the end of these missions, two general surgeons from Ngaoundéré Regional Hospital completed three supervised AVF creation procedures (one radiocephalic, two brachiocephalic). No independent AVF creations by local surgeons without visiting specialist presence were performed during the 2023-2025 period. The vascular surgeon specialist maintained asynchronous communication with local surgeons for post-operative mentoring via the WhatsApp (Menlo Park, CA: Meta Platforms, Inc.) app and phone calls. Standardized AVF management protocol was institutionalized at Ngaoundéré Regional Hospital in January 2025. A vascular access protocol was incorporated into hospital policies and made available to nephrology-hemodialysis and surgery departments. Regarding dialysis nursing training, all nursing staff completed structured training in AVF cannulation techniques, with no cannulation-related complications documented during the first 100 procedures performed by the nurses.

## Discussion

Principal findings and clinical significance

**Figure 4:**
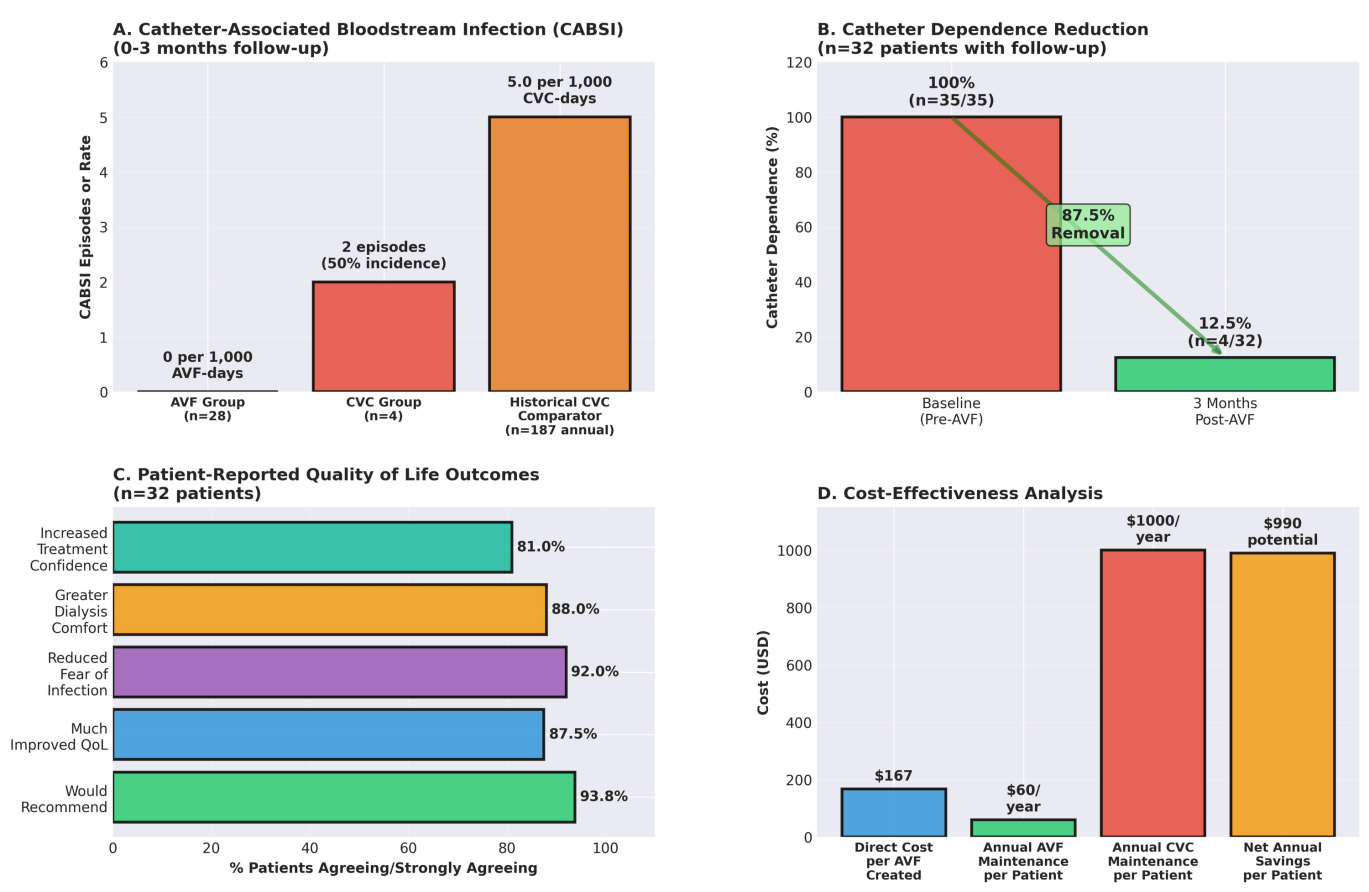
Infection prevention, catheter dependence, patient quality of life, and cost-effectiveness analysis. AVF: arteriovenous fistula; CVC: central venous catheter; QoL: quality of life

This study demonstrates that a time-limited vascular access mission, integrated with point-of-care ultrasound and equity-focused patient prioritization, can achieve functional arteriovenous fistula maturation rates (90.6%, 95% CI: 75.8-96.8%) comparable to reported outcomes in standard settings, despite major infrastructural constraints [18]. Our key findings include surgical feasibility (97.1% of patients underwent successful AVF creation without intra-operative complications), early maturation (90.6% of patients achieved functional, mature AVFs by three months), elimination of vascular access-related infection (zero CABSI in the AVF group versus 5.0 per 1,000 catheter-days in the historical comparator), temporary catheter independence (87.5% of surviving patients discontinued CVC dependence within three months), and high patient satisfaction (92% reported reduced anxiety about infection; 94% would recommend intervention). These outcomes were achieved using local anesthesia, ultrasound instead of angiography, and cost-effective suture materials adapted to local market availability. These results are consistent with reports from other African settings [19]. Synchronization with the World Kidney Day celebrations was essential for success [20]. Point-of-care Doppler ultrasound replaced angiography (typical cost $300-500 per study in sub-Saharan Africa) [21]. Same-day discharge protocols further reduced costs per patient by avoiding hospitalization. This approach aligns with WHO resolutions on point-of-care diagnostics in resource-limited settings [22]. The prioritization framework targeted young, economically disadvantaged patients with potential for long-term dialysis benefit, thereby maximizing the impact per intervention. By enrolling unemployed and underprivileged individuals (51% unemployment rate in the cohort), these missions addressed the structural exclusion of rural patients from life-sustaining health care [23]. The three-month functional patency rate (90.6%) is comparable to published papers [24]. The United States Renal Data System (USRDS) reports 85-92% patency at three months in large cohorts [25]. Our early failure rate (2.9% thrombosis) may be likely attributable to rigorous pre-operative ultrasound selection and standardized surgical technique. A comparison to USRDS benchmarks requires acknowledging major differences. This cohort was younger than the typical US hemodialysis population (median ~65 years), favoring maturation outcomes. Our diabetes prevalence (17.1%) was lower than US rates (50-60%), which may reduce comorbidity-related maturation failure risk [26]. The three-month outcomes represent the minimal scale for maturation assessment per KDOQI guidelines, so we cannot yet comment on long-term primary or secondary patency. Infection prevention, patient satisfaction, catheter independence, and cost-effective analysis are presented in a combined figure (Figures 4A-4D).

Safety profile and complication management

The exclusive use of local anesthesia was safe, underscoring the appropriateness of this choice in dialyzed patients [27]. Early complications, thrombosis, and stenosis were reported but were subsequently corrected. The reduction in catheter-associated bloodstream infection (CABSI) represents a clinically transformative finding for our patients who initially were dependent on non-tunneled CVCs; AVF transition eliminated CABSI entirely in the AVF-using subgroup. This observation in the AVF group aligns with documented epidemiological evidence that AVF substantially reduces the risk of infection compared with CVC [28].

Predictors of maturation failure

Our analysis identified female sex (OR: 7.88, p=0.089), diabetes (OR: 6.25, p=0.077), and small vein diameter <2.5 mm (OR: 13.5, p=0.021) associated with maturation failure. These findings align with published global literature on AVF maturation failure. Systematic reviews have consistently identified female sex, advanced age, and small-caliber vessels as risk factors [29]. Our observations extend these findings to a sub-Saharan African population but should be considered hypothesis-generating rather than definitive, given sample size constraints. While this model demonstrates excellent clinical outcomes during surgical missions, it has not yet established independent, sustainable local vascular surgical capacity. Local surgeons completed only 1.5 AVF procedures per surgeon per year (2023-2025 average), without any independent procedures done by the regional team. Local capacity building would require financial support from national health authorities.

Equity and global health

Beyond clinical results, this vascular mission highlighted inequities in access to life-sustaining care in dialysis patients. The cohort’s average monthly household income ($38) does not facilitate travel to big cities for surgery. An estimated cost of $200-300 for transport + accommodation + fees represented half a year of a patient's income. By eliminating travel barriers and out-of-pocket costs, this model restored patients' dignity. Qualitative feedback revealed psychosocial improvements as follows: 92% reported reduced fear of infection, and 88% noted greater independence in daily functioning. Similar challenges in vascular access care have been reported from other sub-Saharan African dialysis centers. This report mirrors findings from similar initiatives in Malawi and Rwanda, where improved AVF was correlated with social reintegration [30].

Cost-effectiveness: preliminary analysis with important limitations

This is a preliminary, back-of-the-envelope calculation rather than a formal health economic evaluation. A complete cost-effectiveness analysis with quality-adjusted life years (QALYs) or disability-adjusted life years (DALYs) is beyond the scope of this retrospective study but represents a priority for future research. A preliminary cost analysis reveals the economic feasibility of this model. We estimated direct costs per AVF created, based on local realities. The stipend for the visiting vascular surgeon specialist, including travel by plane, accommodation in a mid-range hotel, and three meals a day, was about $67/AVF (mean $800 ÷ 12 cases per mission). The surgical supplies (sutures, drapes, gloves, and antiseptics) cost approximately $45/AVF. Ultrasound mapping (device cost amortized over five years, 150 procedures) gave about $10/AVF. The operating room overhead (utilities, sterilization) was estimated at $25/AVF, and the post-operative follow-up, including four clinic visits with Doppler studies, was about $20/AVF for a total estimated direct cost of $167/AVF. The annual CVC maintenance cost per patient/year, which included catheter kits (replacement every four weeks at $100/kit), related antibiotics (empiric, targeted), and CABSI hospitalizations, exceeded $1000/patient/year at the dialysis center. On the other hand, the AVF maintenance costs per patient/year (Doppler monitoring twice per year at $60/year) are estimated at $60/patient/year, suggesting potential net savings of approximately $990/patient/year.

Important limitations

This retrospective study has several limitations as follows: a small sample size (n=35) that limits statistical power for predictor analysis, wide confidence intervals that reflect sampling variability rather than clinical imprecision, a short three-month follow-up (with 12- to 24-month data collection ongoing), the absence of a concurrent control group for ethical reasons, reliance on unpublished historical audit data for infection rates, the single-center design in a specific geographic and sociocultural context, which limits external validity, uncertain replicability in other African regions, selection bias toward younger and more vulnerable patients who may have better maturation potential, underrepresentation of patients unable to attend follow-up, lack of time-to-event (Kaplan-Meier) analysis, use of a non-validated satisfaction questionnaire, incomplete cost-effectiveness analysis, observer bias (non-blinded nephrologist follow-up), and Doppler-based rather than transit-time flow measurement, which may overestimate flow. Potential temporal differences in care standards between cohorts were considered, but cannot be fully excluded. Nevertheless, as a pilot implementation study, the clinically meaningful improvements remain robust.

## Conclusions

The Ngaoundéré vascular surgery missions demonstrate that access to arteriovenous fistula is feasible for patients in maintenance hemodialysis in remote areas of the African region. To achieve it, equity-focused patient prioritization and a local contextualization are necessary. By closing the vascular access gap without requiring patients to cross borders, this study advocates for all patients to have access to renal care.

## Appendices

Patient satisfaction questionnaire (five-item Likert scale)

This questionnaire was administered orally by a research nurse at the three-month post-operative follow-up visit. Responses were recorded on a five-point Likert scale. The questionnaire was available in French and Fulfulde; the French version is presented below, with English translation provided for publication purposes.

**Table 4:** Patient satisfaction questionnaire (five-item Likert scale). Response scale (1-5): 1 = strongly disagree, 2 = disagree, 3 = neutral, 4 = agree, 5 = strongly agree.

English version	Version française
1. My anxiety about infection has decreased since AVF creation.	1. Mon anxiété liée au risque d’infection a diminué depuis la création de la FAV.
2. My overall quality of life has improved since AVF creation.	2. Ma qualité de vie globale s’est améliorée depuis la création de la FAV.
3. I would recommend this vascular access program to other dialysis patients.	3. Je recommanderais ce programme d’abord vasculaire à d’autres patients dialysés.
4. I am satisfied with my current access to hemodialysis.	4. Je suis satisfait de mon abord vasculaire actuel pour l’hémodialyse.
